# Matrix metalloproteinases in recurrent corneal melting associated with primary Sjörgen’s syndrome

**Published:** 2009-11-14

**Authors:** Kristyna Brejchova, Petra Liskova, Enkela Hrdlickova, Martin Filipec, Katerina Jirsova

**Affiliations:** 1 Laboratory of the Biology and Pathology of the Eye, Institute of Inherited Metabolic Disorders, General Teaching Hospital and Charles University in Prague, 1st Faculty of Medicine, Prague, Czech Republic; 2 Department of Ophthalmology, General Teaching Hospital and Charles University in Prague, 1st Faculty of Medicine, Prague, Czech Republic; 3 Lexum European Eye Clinic, Czech Republic; 4 Ocular Tissue Bank, General Teaching Hospital and Charles University in Prague, 1st Faculty of Medicine, Prague, Czech Republic

## Abstract

**Purpose:**

To investigate the contribution of matrix metalloproteinases (MMPs) to recurrent corneal melting in keratoconjunctivitis sicca associated with primary Sjörgen’s syndrome (pSS).

**Methods:**

One native melted cornea and ten melted corneal grafts from two patients with severe pSS were used. The presence of MMPs (1, 2, 3, 7, 8, 9, and 13) was detected using indirect enzyme immunohistochemistry. The active forms of MMP 2 and 9 and MMP 3 and 7 were examined by gelatin and casein zymography, respectively. The concentrations of active MMP 1 were measured using an activity assay. Eleven unaffected corneas served as controls.

**Results:**

The average values of the staining intensity revealed very intense MMP 1, intense MMP 2, 7, and 9 and moderate MMP 3 and 8 positivity, in the corneal epithelium of melted corneas. Intense MMP 1 and 9 staining, moderate MMP 2, 3, and 8 staining, and weak MMP 7 staining were found in the anterior stroma. The posterior stroma revealed intense MMP 1, moderate MMP 3 and 9, and weak MMP 2, 7, and 8 positivity. Immunostaining of the endothelium was moderate for MMP 9 and weak for MMP 1, 2, 3, 7, and 8. MMP 13 was negative in all but four melted specimens, where weak-to-moderate staining was found in the epithelium and stroma. Control corneas revealed moderate MMP 1 and 2 and weak MMP 8 staining in the epithelium, weak MMP 2 staining in the anterior stroma, and weak MMP 1 and 8 staining in the endothelium. Significantly elevated MMP 1 activity and extremely elevated MMP 9 activity were found in most of the tested pathological specimens, compared to healthy controls, where no activity of the two enzymes was present. Markedly elevated MMP 2 activity was found in 2 of 11 specimens, compared to normal tissue. The inactive form of MMP 3 was detected in half of the tested specimens, and inactive MMP 7 in all melted corneas. Active MMP 3 and 7 were found in one melted sample. Neither of these MMPs was found in any of the control specimens.

**Conclusions:**

The increased expression and elevated activity of a wide range of MMPs in melted cornea samples from two patients diagnosed with pSS confirm that these enzymes contribute to the tissue destruction, leading to serious consequences such as corneal perforation and loss of vision.

## Introduction

Primary Sjögren’s syndrome (pSS) is a systemic autoimmune disease with an estimated prevalence of only about 0.5%. It is characterized by the destruction of the lachrymal and salivary glands, resulting in keratoconjunctivitis sicca syndrome (KCS) and xerostomia [1-3]. There is lymphocytic infiltration in the exocrine glands and the production of various autoantibodies [1,4,5]. Extraglandular systemic manifestations may involve several tissues and organs [3,6-8]. The ethiopathogenesis of pSS is complex; environmental factors are thought to trigger inflammation in individuals with a genetic predisposition, but the exact underlying cause remains unknown [2,5].

Most patients do not exhibit severe ocular complications. Those that need to attend ophthalmology clinics have been found to suffer, in addition to dry eye, from bacterial keratitis, pannus formation, and sterile corneal melting [9-11].

Corneal melting (keratolysis) is a rare, occasionally recurrent condition. It is characterized by the development of epithelial defects and the gradual reduction of stromal components, which may lead to descemetocele formation and subsequent perforation of the cornea [12-15]. Less than 20 cases of sterile corneal melting or corneal ulcers in association with pSS have been described in the literature [11,16,17]. Although the exact mechanism of corneal melting has not been elucidated, it is often linked to the overexpression of matrix metalloproteinases (MMPs), which are considered mainly responsible for the destruction and consequent loss of the extracellular matrix (ECM) [18-21]. Most of these endopeptidases are synthesized as inactive proenzymes that are activated by proteolytic cleavage [22,23]. On the basis of substrate specificity, sequence similarity, and domain organization, MMPs are classified into six groups: collagenases, gelatinases, stromelysins, matrilysins, membrane type MMPs, and others. In the cornea, MMPs can be produced by keratocytes, epithelial cells, monocytes, and macrophages [20,24-27].

In this study, seven MMPs representing four main groups of these endopeptidases were investigated in extremely severe cases of KCS associated with pSS. These included collagenases (MMPs 1, 8, 13) capable of cleaving collagen types I, II, and III [23]; gelatinases (MMP 2 and 9) capable of degrading collagen types IV, V, and VI, as well as decorin, fibronectin, and laminin [28-30]; and stromelysins (MMP 3) and matrilysins (MMP 7), which have similar substrates—type IV collagen, procollagens, collagen cross-links, fibronectin, and laminin [22,23]. We report recurrent corneal melting in two patients with severe pSS and its relation to the activity of major MMPs.

## Methods

The study adhered to the tenets set out in the Declaration of Helsinki. Local Ethics Committee approval was granted. All melted explants were obtained from the Department of Ophthalmology, General Teaching Hospital and the 1st Faculty of Medicine, Charles University, in Prague.

### Case report 1

A 77-year-old patient was diagnosed with pSS elsewhere at the age of 71. She tested positive for anti-SS-A/Ro, anti-SS-B/La, and antinuclear antibodies. No extraglandular manifestations were noted. Systemic immunosuppression administered to the patient included various combinations of cyclophosphamide, prednisolon, azathioprin, methylprednisolon, cyclosporine A, and mycophenolate mofetil. Upon first examination in our Department of Ophthalmology in 2002 at age 74, the patient presented with bilateral severe dry eye syndrome. In the course of three years, she suffered from numerous episodes of corneal melting, requiring a number of surgical procedures. In the right eye, seven penetrating keratoplasties were performed (6 grafts were used as specimens S2, S3, S4, S6, S7, and S8), along with a number of amniotic membrane transplants, tarsorrhaphies, and other surgeries aimed at improving the condition of the ocular surface and preventing progressive tissue melting. In the left eye, the patient underwent four penetrating keratoplasties (her native cornea was used as specimen S1 and one graft as specimen S5), as well as other, similar, surgeries to the right eye. Despite all the measures undertaken, her condition could not be controlled, and it led to bilateral blindness.

### Case report 2

In case 2, the symptoms related to pSS started at the age of 46 when swollen salivary glands, xerostomia, and severe dry eye symptoms were noted by the patient. Histopathology from a labial salivary gland biopsy sample showed focal sialadenitis that was consistent with a diagnosis of pSS. The patient tested positive for rheumatoid factor, antinuclear antibodies, and Scl70 antibodies. Subsequently she also developed arthralgias. Systemic immunosuppression therapy was started at the age of 50. Initially, the patient was treated with oral prednisolon; later, various combinations of methotrexate, cyclophosphamide, and methylprednisolon were added. Upon first ocular examination at age 46, she had signs of moderate dry eye syndrome in both eyes. She gradually developed severe dry eye syndrome bilaterally. When the patient reached the age of 58, the first signs of peripheral ulcerative keratitis were observed in the right eye, followed three years later in the left eye. She rapidly developed corneal thinning, and underwent her first keratoplasty in the right eye at the age of 59 (specimen S9), followed by numerous other procedures due to complications related to melting of the graft, including two penetrating keratoplasties (specimens S10 and S11). At the last follow up visit, her visual acuity was full light projection in the right eye and hand movements with full projection of light in the left eye.

### Specimen preparation

Eleven melted corneal specimens of the two patients were processed within three h after surgery. Eleven unaffected donor corneal buttons (mean age 59.8±16.9 years) that were unsuitable for transplantation, due to their low endothelial cell density, served as controls (obtained from the Ocular Tissue Bank, General Teaching Hospital, Prague). The mean time from the donor’s deaths to enucleation was 15 h, and the mean time from death to tissue freezing was 17 h. All specimens were dissected into two halves, snap-frozen in liquid nitrogen, and stored at 70 ºC. Prior to freezing, one-half was embedded in Optimal Cutting Temperature Compound (Christine Gröpl, Tulln, Austria). Before the activity assessment, the specimens were thawed, were homogenized in cacodylate buffer (0.1 M cacodylic acid, 0.15 M NaCl, 0.01 M CaCl_2_, 1.5 mM NaN_3_, 0.005% Triton X-100, and 0.1 nM ZnCl_2_), and underwent protein extraction for 2 days at 4 °C. Next, they were centrifuged for 30 min at 10,000× g; the supernatants were removed and frozen at -20 °C.

### Indirect enzyme immunohistochemistry

Cryosections (7 μm thick) from each of the control and melted specimens were placed on gelatin-coated glass slides (four per slide), fixed with cold acetone for 10 min, rinsed in phosphate-buffered saline (PBS), and incubated for 30 min in 3% hydrogen peroxide in PBS. After washing in PBS, the specimens were blocked for 30 min with 2.5% bovine serum albumin in PBS. Then the slides were incubated for 1 h at room temperature with the primary antibodies listed in Table 1. Subsequently, the slides were washed in PBS, and the secondary antibodies (polyclonal rabbit anti-mouse IgG and swine anti-rabbit IgG conjugated with biotin, 1:200; DakoCytomation, Glostrup, Denmark) were applied for 1 h. After rinsing in PBS (three times for 5 min each), streptavidin/HRP (1:250; DakoCytomation, Glostrup, Denmark) was added for 30 min. Finally, the slides were developed with 0.06% 3,3'-diaminobenzidine tetrahydrochloride (Fluka, Buchs, Switzerland) in PBS, counterstained with Harris hematoxylin, and mounted with Eukit (Fluka, Buchs, Switzerland). Negative control specimens (primary antibody omitted) were included on each slide. Samples of human placenta (MMP 1, 2, 3, and 7) and breast carcinoma (MMP 7, 8, 9, and 13) were used as positive controls [31,32]. The intensity of the signal was assessed separately in the epithelium, anterior stroma, posterior stroma, and endothelium using five grades of positivity: 0 (negative), 1 (weak), 2 (moderate), 3 (intense), 4 (very intense). The mean average positivity was calculated from at least three sections of two independent experiments.

**Table 1:** Matrix metalloproteinase detecting antibodies used for indirect immunohistochemistry.

**Antibody**	**Catalogue number**	**Concentration**	**Company**
Polyclonal rabbit anti-human MMP 1	AB8105	1:300	Chemicon Intl. Inc.
Monoclonal mouse anti-human MMP 2	MAB13431	1:350	Chemicon Intl. Inc.
Polyclonal rabbit anti-human MMP 3	29576	1:50	AnaSpec Inc., San Jose, CA
Monoclonal mouse anti-human MMP 7	MAB13414	1:150	Chemicon Intl. Inc.
Polyclonal rabbit anti-human MMP 8	AB8115	1:300	Chemicon Intl. Inc.
Monoclonal mouse anti-human MMP 9	MAB3309	1:150	Chemicon Intl. Inc.
Monoclonal mouse anti-human MMP 13	MAB13424	1:50	Chemicon Intl. Inc.

### Gelatin and casein substrate zymography

All specimens (native cellular protein quantity, 8.5 μg) were treated with sample buffer (1.5% sodium dodecyl sulfate [SDS], 15% glycerol, and 0.005% bromphenol blue). Gelatin and casein zymography were performed for the detection of MMP 2 and 9, and MMP 3 and 7, respectively, using 10% polyacrylamide gel containing 0.1% gelatin (AppliChem GmbH, Darmstadt, Germany) and 12% gel copolymerized with 0.09% β-casein (Sigma-Aldrich, St. Louis, MO). In brief, 20 μl of each specimen were loaded onto the gels, and SDS-polyacrylamide gel electrophoresis (SDS-PAGE) was performed at 200 V at 4 °C for gelatin zymography, and at 20 mA at 4 °C for casein zymography. After electrophoresis, the gels were twice rinsed in 2.5% Triton X-100 for 30 min at room temperature to remove the SDS, then incubated in reaction buffer (50 mM Tris-HCl, pH 7.5; 200 mM NaCl,; 5 mM CaCl_2;_ and 0.02% 23 lauryl ether[Brij-35]) at 37 °C overnight to allow the proteinases to digest their substrates. The gels were stained for 1 h at room temperature in 0.5% Coomassie brilliant blue R-250 (Serva Electrophoresis, Heidelberg, Germany) in 40% methanol and 10% acetic acid, then destained with a mixture of 40% methanol and 10% acetic acid. Proteolytic activities appeared as clear bands of lysis against a dark background of stained gelatin or casein.

### Matrix metalloproteinase 1 activity assays

The concentrations of the active forms of MMP 1 were determined using a commercial kit (Amersham matrix metalloproteinase-1 Biotrak Activity assay system, Amersham Biosciences, Amersham, UK) according to the manufacturer’s protocol. The values of the color reaction of the assays were read at 405 nm in a SUNRISE ELISA Reader (Tecan Trading AG, Männedorf, Switzerland). The activity of MMP 1 in ten samples was determined by interpolation from the standard curve. The activity assay could not be performed in S3, due to the lack of sufficient specimens.

### Statistical analysis

The Mann-Whitney U test was used to analyze the differences between the control and the experimental groups. A p value <0.05 was considered to indicate statistical significance.

## Results

### Localization of individual matrix metalloproteinases in melted and control specimens

All control corneas exhibited regular morphology, with a five- to six-layered epithelium. Severe damage was observed in most of the pathological specimens, ranging up to the complete absence of the epithelial layers and, in some specimens, a partly dissolved Bowmann’s layer and a partly dissolved edge of the anterior stroma in the area of the lesions.

**Figure 1:**
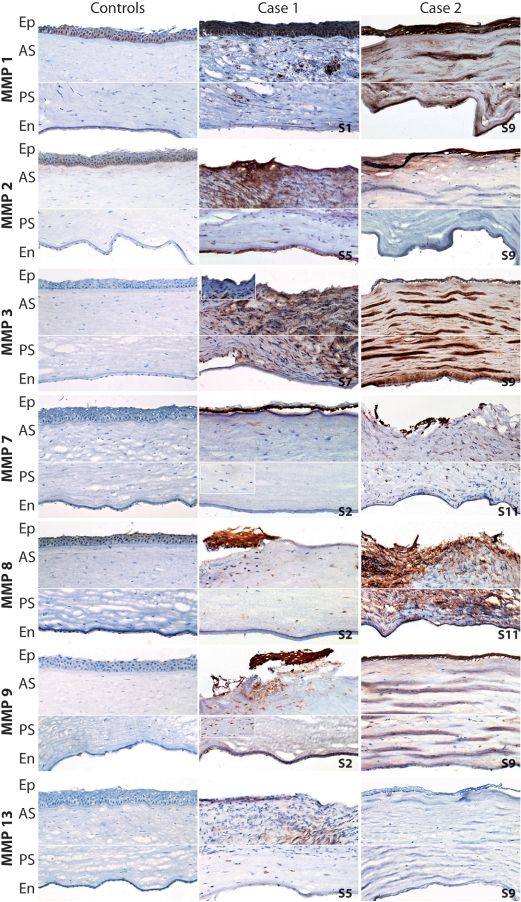
Immunohistochemical localization of matrix metalloproteinases in melted and control corneas. Immunohistochemical localization of MMP 1, 2, 3, 7, 8, 9, and 13 in representative images of melted corneal specimens obtained from patients with pSS (cases 1 and 2) and from control corneas. Original magnification, 100×. Ep = epithelium, AS = anterior stroma, PS = posterior stroma, En = endothelium.

The levels of staining of antibodies against particular MMPs in control samples were averaged. No prominent differences in MMP staining were found among the individual control specimens for any of the MMPs tested. Moderate staining for MMP 1 and 2, and weak staining for MMP 8, were detected in the epithelium. A weak signal for MMP 2 was observed adjacent to Bowman’s layer, in approximately one-sixth of the anterior stroma. Weak staining was also found for MMP 1 and 8 in the endothelium of the control specimens. Immunostaining for MMP 3, 7, 9, and 13 was completely negative in all layers of all control corneas (Figure 1).

The staining intensity of different MMPs in melted specimens is summarized in Table 2. Immunohistochemical staining of both the control and melted specimens is shown in Figure 1. In the melted specimens, stronger staining for MMP 9 was found in the epithelial fragments of all tested corneas, if they were not destroyed. It was also found for MMP 1, 2, 3, and 7 in almost all melted corneas, compared to the controls. Increased staining for MMP 1 and 9 was found in the whole stroma in all tested corneas, and for MMP 2, 3, 7, and 8 in almost all melted grafts, compared to the controls. Positivity staining for MMP 2, 3, and 7 was detected in the endothelium of a few melted specimens, while staining for MMP 9 was observed in all melted grafts. MMP 13 revealed only a weak-to-moderate staining pattern in the epithelial fragments and in the stroma of four specimens and in the endothelium of one specimen.

**Table 2:** The immunohistochemical localization of individual MMPs in the corneal specimens obtained from two patients with pSS (S1-8 and S9-11 respectively) and the average values of immunohistochemical staining in all melted specimens (S) and controls (C).

**MMP**	**Corneal layer**	**Sample**												
		**S1**	**S2**	**S3**	**S4**	**S5**	**S6**	**S7**	**S8**	**S9**	**S10**	**S11**	**S**	**C**
MMP 1	Ep	4	4	D	D	D	2	D	4	4	D	D	4	2
	AS	3	4	3	2	3	1	4	4	4	2	3	3	0
	PS	2	3	2	2	4	2	3	4	4	1	3	3	0
	En	0	3	D	D	0	0	D	D	2	D	D	1	1
MMP 2	Ep	3	3	D	D	D	4	3	2	4	D	D	3	2
	AS	4	2	2	0	4	2	2	3	3	2	3	2	1
	PS	3	2	1	0	1	0	0	3	0	2	1	1	0
	En	2	1	0	0	3	2	0	D	1	0	0	1	0
MMP 3	Ep	2	2	D	D	D	2	0	2	3	2	3	2	0
	AS	2	2	1	0	2	1	4	2	4	0	2	2	0
	PS	1	0	2	0	2	1	4	2	4	0	2	2	0
	En	D	0	0	0	1	0	D	D	2	D	D	1	0
MMP 7	Ep	0	4	D	D	D	4	3	4	0	D	4	3	0
	AS	0	1	2	0	3	2	1	3	0	0	2	1	0
	PS	0	1	2	0	3	1	1	3	0	0	2	1	0
	En	D	D	D	0	2	1	D	D	0	D	D	1	0
MMP 8	Ep	2	4	D	D	D	1	0	2	2	D	D	2	1
	AS	2	1	1	2	3	2	0	2	1	2	4	2	0
	PS	0	1	0	2	3	1	0	2	1	1	4	1	0
	En	D	D	0	D	2	0	0	D	2	0	D	1	1
MMP 9	Ep	2	4	D	D	D	1	2	2	4	D	3	3	0
	AS	3	3	1	3	4	4	2	3	1	2	3	3	0
	PS	1	3	0	1	2	3	2	3	1	1	4	2	0
	En	4	2	D	1	2	1	D	D	1	D	0	2	0
MMP 13	Ep	0	0	D	D	D	1	0	1	0	D	D	0	0
	AS	0	0	1	0	2	1	0	1	0	0	0	0	0
	PS	0	0	1	0	2	0	0	1	0	0	0	0	0
	En	D	0	0	0	1	0	D	D	0	0	D	0	0

The scale used for the intensity of the signal: 0 - negative, 1 - weak, 2 - moderate, 3 - intense, 4 - very intense positivity. D = destroyed tissue. Ep = epithelium, AS = anterior stroma, PS = posterior stroma and En = endothelium.

### Detection of metalloproteinase activity

#### Gelatin and casein zymography

**Figure 2:**
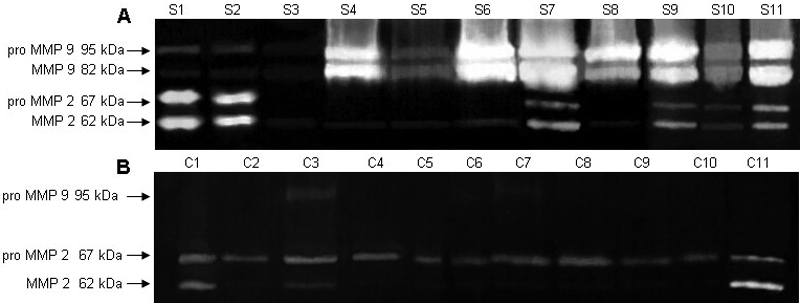
Gelatin zymography of matrix metalloproteinases 2 and 9 in melted and control corneas. Melted specimens **(A) **S1 and S2 showed extremely high levels, and specimens S7, S9, and S11 considerable levels, of both the proenzyme (67 KDa band) and the active form of MMP 2 (62 kDa band). Levels of MMP 9 proenzyme (95 kDa band) and the active form (82 kDa band) were extremely high in S4 and S6-S11, and prominent in S5. Weak bands for both MMP 9 forms were found in specimens S1-S3. In control samples **(B)**, a moderate level of the MMP 2 proenzyme was present in all specimens, whereas the active form of MMP 2 was either not present or very faint, except in samples C1, C3, and C11. As for MMP 9 proenzyme, only C3 and C7 showed faint bands.

Using gelatin zymography, 2 of the 11 melted specimens (S1 and S2) displayed extremely high levels of both the proenzyme and active form of MMP 2, compared to the control corneas. Levels of MMP 9 proenzyme and the active form were extremely high in seven (S4, S6-S11) melted specimens and prominent in one (S5). Three melted specimens (S1-S3) revealed faint bands for both forms of MMP 9, and two controls did so for MMP 9 proenzyme only (Figure 2A).

**Figure 3:**
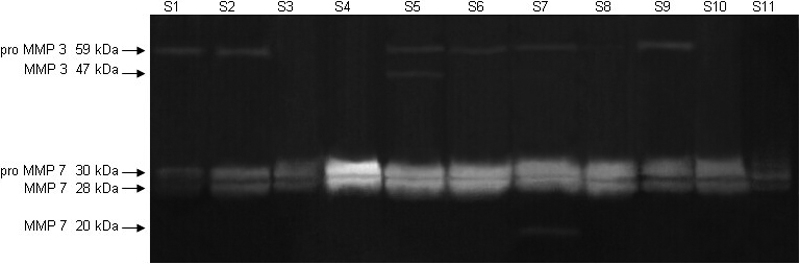
Casein zymography of matrix metalloproteinases 3 and 7 in melted specimens. Very slight bands of only the MMP 3 proform (59 kDa) were detected in five melted specimens (S1, S2, S6, S7, and S9) and of both the proform and active MMP 3 (47 kDa) in one specimen (S5). High levels of MMP 7 proenzyme (30 kDa) and its intermediate cleavage product (28 kDa) were detected in nine melted corneas, S2-S10, and weak bands of these two MMP 7 inactive forms were found in two melted specimens (S1 and S11). A very slight band of active MMP 7 (20 kDa) was found in one specimen only (S7).

Casein zymography revealed neither the proenzyme nor the active enzyme of MMP 3 or 7 in any of the control specimens. Negligible levels of the proform of MMP 3 were found in five melted specimens (S1, S2, S6, S7, and S9). Both forms, the proform and active MMP 3, were detected in one sample (S5) only. Nine melted corneas (S2-S10) revealed high levels of MMP 7 proenzyme and its intermediate cleavage product. Two samples (S1 and S11) revealed low levels. Very low levels of active MMP 7 were found in one specimen (S7) only (Figure 3).

#### Matrix metalloproteinase 1 activity assay

The active form of MMP 1 was found in eight of ten melted corneas at concentrations ranging from 0.08 to 3.03 ng/ml (p=0.0011). No activity was detected in the control specimens (Table 3).

**Table 3:** MMP 1 Activity Assay. The concentrations of the active forms of MMP 1 in melted corneal tissue were determined by interpolation from the standard curve.

**Specimen**	**Concentration of active MMP 1 (ng/ml)**
S1	0.69
S2	0.08
S4	0.23
S5	0.7
S6	0.23
S7	0.0
S8	0.39
S9	0.0
S10	3.03
S11	1.22

## Discussion

In this study, we present two cases with pSS undergoing rapidly progressive recurrent corneal melting despite all available treatment, including immunosuppression. We obtained two series of tissue specimens from the eyes of one repeatedly grafted patient: six consecutive ones from the right eye, and two from the left . We also obtained one series of three consecutive tissue samples from the right eye of another patient with pSS. Our findings clearly demonstrated the increased presence of MMP 1, 2, 3, 7, 8, and 9, as well as higher activity of MMP 1, 2, 3, 7, and 9, in the pathological pSS specimens, compared to the control tissue.

To the best of our knowledge, this is the first time that these enzymes have been studied in corneal melting associated with pSS. Previously, differences in corneal MMP expression were detected in patients with keratolysis associated with rheumatoid arthritis [21], an autoimmune disorder that has some overlapping clinical features with pSS, and in patients with melted corneas after cataract surgery and photorefractive keratectomy, both of which are treated with diclophenac [18,19,33]. Our study demonstrated a statistically significant higher activity of MMP 1 and a high expression of MMP 9 in the corneal epithelium and stroma. Both these results conform to the immunostaining results of others [18,19,21,33]. As for MMP 2, its overexpression in both the epithelium and stroma, as well as its higher activity, have been reported in other investigations [18,33]. However, one study showed only a weak presence of MMP 2 in the stroma of a patient who had undergone cataract surgery and had been treated with diclophenac [19]. Our results also demonstrated variability in the MMP 2 expression in melted corneas, since only 2 of the 11 specimens revealed a considerable increase in its activity, compared to the controls. One possible explanation for this phenomenon is that MMP 2 activity is limited to a short period in the melting process, unlike the other MMPs. This hypothesis is supported by delays in the activity increase of this enzyme in corneas after alkali burn, suggesting its role to be in the regeneration and remodeling of the corneal ECM, rather than in the degradation process [34,35].

A marked increase of MMP 3 in the epithelium and stroma of melted grafts has been detected previously, in the stroma of a patient after photorefractive keratectomy treated with topical diclofenac [18]. Although we confirmed the presence of MMP 3 in melted corneas as well, we were not able to detect a prominent increase in its activity. This may be due to the low sensitivity of casein zymography [36]. Unfortunately, there was not enough material available to perform other, more sensitive methods of MMP 3 detection.

Our study is the first to demonstrate the presence and activity of MMP 7 in melted corneas. Additionally, casein zymography showed a large quantity of inactive MMP 7 in all tested specimens and active MMP 7 in one specimen. We suggest that MMP 7 is an important element in the degradation of the corneal basement membrane in corneal melting, as it was abundant in the corneal epithelium, especially in its basal layer.

Up to now, MMP 8 has been studied only in one melted cornea following cataract surgery, where it was found to be considerably increased in both the epithelium and stroma [19]. In our study, we found a weak-to-moderate presence of this enzyme in both the epithelium and stroma. We attribute such differences to the fact that the occurrence of MMP 8 in the stroma depends on the presence of neutrophils [22], the distribution of which may vary among melted corneas [19,37,38].

We also found a weak increase in the presence of MMP 13 in the stroma of three samples. To the best of our knowledge, no other study has previously evaluated MMP 13 in melted corneas. The expression of MMP 13 has only been described in the epithelium of wounded corneas [39] and in the epithelium and stroma of corneas with keratoconus [40].

We did not observe any trend towards an increase or decrease of individual MMP expression over time, or of disease progression, in any of the consecutive patient samples. Instead, the combination of MMPs detected seemed to be completely different in each specimen. This could have a number of causes, such as the different stages of melting at which the explants were obtained. It may also be that the expression of MMPs showed local variations within individual specimens, depending on the distance from the central melting point. It should be noted, however, that the staining and activity of individual MMPs were similar for consecutive sections obtained from each specimen.

MMPs in patients suffering from primary SS have previously been studied in tears [41], saliva [42], and salivary glands [43,44]. It has been suggested that the activation of these enzymes is the key factor responsible for the corneal barrier disruption, as well as for the destruction of the salivary glands [42,44-46]. Given the characteristic features of pSS, there may be more than one mechanism leading to the induction of different MMPs. For example, lymphocytic infiltrates secrete pro-inflammatory cytokines [2] that are known to initiate MMP expression in various tissues via different pathways [22]. Up-regulated IL-1β, found in the tears of pSS patients [41], could also play a role in the expression of MMPs, especially MMP 9, via mitogen-activated protein kinase signaling pathways [47]. Finally, a mouse model of dry eye has shown that desiccation and hyperosmolar stress may lead to the induction of MMPs via the stimulation of proinflammatory cytokines [45,47]. We hypothesize that in advanced cases of pSS, such as in our patients, MMPs may be upregulated to such an extent that the epithelial barrier is substantially degraded, followed by the dissolution of its basement membrane (caused mainly by MMP 3, 7, and 9) and the gradual degradation of the stroma, involving MMP 1, 3, 7, 8, and 9. After the stroma is completely lost, a descemetocele is formed, and finally the integrity of the whole cornea is disrupted.

The fact that none of the disease-modifying therapies used in these patients was effective in decreasing MMP production and keratolysis suggests that different treatment strategies with anti-MMP therapies should be considered in similar cases, such as using recombinant tissue inhibitors of MMPs [48] or chemical inhibitors of MMPs. For example, the TNF-α antagonist infliximab has been shown to inhibit one of the activators of MMP production [49]. Direct inhibition of MMPs can be achieved by tetracyclines, medroxyprogesteron, or ion-chelating agents such as cysteine or thylenediaminetetraacetic acid [50-53]. Finally, an alternative approach in keratolysis treatment could be focused on the recovery and strengthening of the collagen structure by collagen cross-linking [54].

Our study examined extremely severe cases of corneal melting associated with pSS, and has elucidated the participation of some MMPs in this destructive process. It confirmed that these enzymes play an important role in the severe degradation of corneal tissue leading to corneal perforation and loss of vision. Their involvement suggests that MMP inhibitors may play an important role in the treatment of this condition.
